# *Campylobacter jejuni* Serine Protease HtrA Induces Paracellular Transmigration of Microbiota across Polarized Intestinal Epithelial Cells

**DOI:** 10.3390/biom12040521

**Published:** 2022-03-30

**Authors:** Irshad Sharafutdinov, Nicole Tegtmeyer, Mathias Müsken, Steffen Backert

**Affiliations:** 1 Department of Biology, Division of Microbiology, Friedrich-Alexander-Universität Erlangen-Nürnberg, Staudtstr. 5, D-91058 Erlangen, Germany; irshad.sharafutdinov@fau.de (I.S.); nicole.tegtmeyer@fau.de (N.T.); 2 Central Facility for Microscopy, Helmholtz Centre for Infection Research, Inhoffenstraße 7, D-38124 Braunschweig, Germany; mathias.muesken@helmholtz-hzi.de

**Keywords:** *Campylobacter*, serine protease, E-cadherin, *Escherichia coli*, HtrA, *Lactococcus lactis*, tight junction, microbiota, commensal

## Abstract

*Campylobacter jejuni* represents an eminent zoonotic germ responsible for foodborne infections causing campylobacteriosis. In addition, infections with *C. jejuni* constitute a risk factor for the occurrence of inflammatory bowel disease (IBD). In the latter case, patients show inflammatory reactions not only against *C. jejuni,* but also against the non-infectious microbiota. However, the involved mechanisms and molecular basis are still largely unclear. We recently reported that *C. jejuni* breaches the intestinal epithelial barrier by secretion of serine protease HtrA (high temperature requirement A), which cleaves several major tight and adherens junction proteins. In the present study, we aimed to study if HtrA-expressing *C. jejuni* may also trigger the transepithelial migration of non-pathogenic gastrointestinal microbiota. Using confocal immunofluorescence and scanning electron microscopy, we demonstrate that *C. jejuni* wild-type (wt) as well as the isogenic ∆*htrA* mutant bind to the surface of polarized intestinal Caco-2 epithelial cells, but do not invade them at the apical side. Instead, *C. jejuni* wt, but not ∆*htrA* mutant, disrupt the cellular junctions and transmigrate using the paracellular route between neighboring cells. Using transwell filter systems, we then co-incubated the cells with *C. jejuni* and non-invasive microbiota strains, either *Escherichia coli* or *Lactococcus lactis*. Interestingly, *C. jejuni* wt, but not ∆*htrA* mutant, induced the efficient transmigration of these microbiota bacteria into the basal compartment. Thus, infection of the intestinal epithelium with *C. jejuni* causes local opening of cellular junctions and paracellular translocation in an HtrA-dependent manner, which paves the way for transmigration of microbiota that is otherwise non-invasive. Taken together, these findings may have impacts on various *Campylobacter*-associated diseases such as IBD, which are discussed here.

## 1. Introduction

*Campylobacter (C.) jejuni* is a leading zoonotic disease-causing agent and the primary cause of bacterial gastroenteritis transmitted through food worldwide, with more than 96 million cases every year [1]. Commonly, these bacteria asymptomatically inhabit the lower intestinal tract of many animals. However, *C. jejuni* can result in serious disease conditions when infecting humans [2,3]. The primary transmission route of the bacterium to humans occurs during the consumption of improperly cooked poultry meats. A recent meta-analysis study reported the mean occurrence rate of *C. jejuni* in broiler meat to be 33.7% worldwide [4]. Defeathering and exenteration have been detected as the most critical points that can seriously influence the microbial security of the foodstuff [5]. Typically, *C. jejuni* infections can last for up to two weeks and can be self-limited by healthy individuals, while complications mostly appear in children, elderly or immunocompromised persons. As a possible long-term sequelae, campylobacteriosis may rarely result in autoimmune diseases, including Miller Fisher or Guillain–Barré syndromes [6], reactive arthritis [7] and inflammatory bowel disease (IBD) [8]. IBD describes a chronic inflammatory disease of the gastrointestinal system, probably resulting from aberrant host immune reactions against the microflora, but its molecular basis is still not fully understood [9].

Usually found in the intestine, *C. jejuni* have been also isolated from other organs including spleen, mesenteric lymph nodes [10], liver [11] and blood [12]. Therefore, *C. jejuni* obviously has capabilities to infect the gastrointestinal tract and to travel into other organs of the host. However, the exact mechanisms allowing this plasticity in migration still remain widely unclear. Numerous virulence-associated determinants provide *C. jejuni* pathogenicity-associated survival, motility, attachment, invasion, cellular transmigration and access to deeper tissue [13]. There is evidence that the bacterial virulence factor HtrA (high temperature requirement A) has a major function in the transmigration of *C. jejuni*. HtrA family proteins are widely distributed in prokaryotes and eukaryotes and perform either proteolytic or chaperone activities [14,15]. Bacterial HtrA belonging to the serine protease family exhibit a signal peptide at the n-terminus, a trypsin-like serine protease domain as well as one or two c-terminal postsynaptic density 95/discs large/zonula occludens-1 (PDZ)-domains [16,17]. Importantly, HtrA family proteins play a crucial part in disease development of many Gram-negative and Gram-positive bacterial pathogens [15]. In particular, *C. jejuni* is able to secrete HtrA into the extracellular environment. On average, various strains obtained comparable high secretion values of HtrA, with strain 81–176 secreting 5483 ± 1246 and strain 11168 secreting 4314 ± 949 molecules per bacterium after 2 h in liquid growth medium [18]. Secreted HtrA can then cleave the host cell junction components claudin-8, occludin and E-cadherin [19,20,21,22]. Interestingly, an occludin gene knockout produced by clustered regularly interspaced short palindromic repeats/Cas9 (CRISPR/Cas9) in intestinal Caco-2 cells disturbed the cell polarity and reduced the transepithelial electrical resistance (TER) compared to wild-type control cells [22]. In addition, occludin deletion permitted higher numbers of *C. jejuni* to travel through the tight junctions. Therefore, disrupted junctions open up a transmigration route between neighboring cells that allows *C. jejuni* to easily reach basal layers of the intestinal epithelium. Moreover, a crucial role of HtrA in pathogenesis of *C. jejuni* was also verified in various murine models [23,24,25,26]. In addition, *C. jejuni* appears to have a great impact on the crosstalk between host and gut microbiota, likely by affecting the integrity of intestinal epithelia. Interestingly, mice seriously affected by intestinal inflammation as well as *C. jejuni*-susceptible infant mice have shown specific microbiota shifts with elevated amounts of commensal *Escherichia coli* [27].

The microbiota, composed of different microorganisms, including commensal bacteria, colonizes the human gut and was shown to greatly influence digestive functions, but also the host immune system [28]. Consequently, it has been shown that intestinal dysbiosis may result in overrepresentation of some putative pathogenic bacteria and promote chronic inflammation [29]. In particular, an increase in the population of some proteobacterial members has strong associations with the progression of Crohn’s disease and ulcerative colitis—two forms of IBD [30]. Two major proteobacteria, *E. coli* and *C. jejuni*, have been shown to promote IBD, however, the molecular mechanism remained widely unclear [8,31,32]. It was suggested that *C. jejuni* possibly facilitate transmigration of non-invasive *E. coli* through a transcellular pathway [33] or a paracellular pathway by disrupting tight junctional claudin-4 [34]. Nevertheless, the bacterial factor responsible for the disruption of tight junction components in order to facilitate the transmigration of other bacteria is still unknown. In this work, we studied if HtrA-expressing *C. jejuni* may trigger the transepithelial migration of non-pathogenic gastrointestinal microbiota strains such as *E. coli* or *Lactococcus lactis*. Here, we explicitly show that *C. jejuni* infection facilitates the effective paracellular transmigration of microbiota strains, and this scenario strongly depends on the protease HtrA.

## 2. Materials and Methods

### 2.1. Cultivation of Bacterial Strains

The *C. jejuni* wild-type (wt) isolate 81–176 and its isogenic chloramphenicol-resistant *htrA*-deficient mutant (Δ*htrA*) were utilized in this work [19,35]. For the co-infection experiments with *C. jejuni* wt and *E. coli* or *L. lactis*, we used a streptomycin-resistant variant of 81–176 for selection, kindly provided by David Hendrixson (University of Texas Southwestern, Dallas, TX, USA). For *C. jejuni* cultivation, plates with Campylobacter blood-free selective agar base and CCDA selective supplement (Oxoid, Wesel, Germany) were used [36]. In the case of antibiotic-resistant strains, we added 20 μg/mL chloramphenicol or 10 μg/mL streptomycin (Sigma-Aldrich, St. Louis, MO, USA). The bacteria were incubated in jars for two days at 37 °C under microaerobic conditions produced by CampyGen^TM^ pouches (Oxoid, Wesel, Germany) [37]. *E. coli* strain HB-101 (Promega GmbH, Walldorf, Germany) was grown on standard Luria broth (LB) agar plates for 16 h, and *L. lactis* (NCTC 6681) was cultivated for 48 h at 37 °C on MRS (de Man, Rogosa and Sharpe; Carl Roth, Karlsruhe, Germany) agar plates in a conventional incubator. For collection and resuspension of all plate-grown bacterial strains, sterile cotton swabs and liquid LB broth were used. To quantify the number of bacterial cells, we applied an Eppendorf spectrophotometer (Eppendorf, Hamburg, Germany) adjusted to a wavelength of 600 nm for measuring the optical density (OD).

### 2.2. Cultivation of Caco-2 Cells Used for Infection Assays

Human intestinal Caco-2 cells (American Type Culture Collection HTB-37™) were cultivated in T75 tissue culture flasks and, for infection, the cells were subcultured in 12-well plates using Dulbecco’s Modified Eagle Medium (DMEM) with high glucose and pyruvate supplemented with 10% FCS (both from Invitrogen, Waltham, MA, USA). Caco-2 cells were seeded in confluent single cell layers and further grown for 2 weeks to enable appropriate polarization of the cells, followed by host cell infection. For this purpose, we added the bacteria to the cells using multiplicities of infection (MOI), as indicated in the figure legends, followed by co-incubation for different time points. Afterwards, pre-warmed PBS buffer (Sigma-Aldrich, St. Louis, MO, USA) was used to wash the infected cells two times, followed by preparation for microscopy (Leica Microsystems, Wetzlar, Germany and Zeiss, Oberkochen, Germany respectively) as described below.

### 2.3. Transwell System and Measurement of Transepithelial Electrical Resistance (TER)

Caco-2 cells were cultivated in cell culture inserts (0.33 cm^2^) with 3 μm pore size (Merck Millipore, Darmstadt, Germany) for 14 days as described above. TER measurement was performed using the Electrical Resistance System (ERS) (Merck Millipore, Burlington, MA, USA). To calculate the TER values (given in Ohms x cm^2^), the fluid resistance was subtracted, and the monolayer surface area was multiplied. The obtained maximum TER values revealed the integrity of the cellular barriers and achievement of maximal cell polarity. Infection of the Caco-2 cells with the bacteria was carried out as described above in a time course up to 8 h. The number of colony-forming units (CFU) in the bottom chamber was counted by bacterial growth on Campylobacter agar, MRS or LB plates. To avoid cross-contamination during CFU determination, we confirmed that *C. jejuni* cannot grow on MRS or LB plates under aerobic conditions in the incubator, and that *E. coli* and *L. lactis* cannot grow on the streptomycin- or chloramphenicol-containing Campylobacter agar plates.

### 2.4. Scanning Electron Microscopy

Caco-2 cells were grown on glass cover slips (12 mm diameter) in a similar fashion as mentioned above. Uninfected Caco-2 cells or cells infected for 6 h by *C. jejuni* were fixed for one hour at 4 °C in HEPES buffer (0.1 M HEPES, 0.09 M sucrose, 0.01 M CaCl_2_, 0.01 M MgCl_2_, pH 6.9) including 5% formaldehyde and 2% glutaraldehyde [38], followed by two washing steps with TE buffer (TRIS 10 mM, EDTA 2 mM, all reagents from Sigma-Aldrich, St. Louis, MO, USA). Afterwards, dehydration of the samples was performed using a graded series of acetone (10%, 30%, 50%, 70% and 90%, Carl Roth, Karlsruhe, Germany) for 10 min at each step, followed by incubating the samples in 100% acetone twice at room temperature. The automated CPD300 dryer (Leica Microsystems, Wetzlar, Germany) was used to carry out critical point drying. Afterwards, cover slips were mounted on 12 mm aluminum stubs with Leit adhesive carbon tabs and sputter-coated with gold/palladium in the SCD 500 (Bal-Tec, Balzers, Lichtenstein). To analyze the samples, a Merlin field emission scanning electron microscope (Zeiss, Oberkochen, Germany) was applied at an acceleration voltage of 5 kV, an inlens-Se detector/Everhart-Thornley SE detector ratio of 75:25 and optimized contrast and brightness settings.

### 2.5. Confocal Immunofluorescence Staining

Immunofluorescence staining was performed as reported earlier [39]. Briefly, the infected cells of interest were washed two times with pre-warmed PBS buffer (Sigma-Aldrich, St. Louis, MO, USA) to eliminate unattached bacteria followed by fixation for 30 min in 4% paraformaldehyde (PFA, Sigma-Aldrich, St. Louis, MO, USA) at room temperature. Subsequently, 0.25% Triton-X100 was used to permeabilize the fixed cells for 10 min followed by blocking for 1 h in PBS buffer supplemented with 3% BSA (all reagents from Carl Roth, Karlsruhe, Germany). Immunostaining of the tight junctions was performed using either rabbit α-occludin (#42-2400) or FITC-conjugated mouse α-occludin (#331588, both from Invitrogen, Waltham, MA, USA) antibodies. Staining of *C. jejuni* bacteria was carried out by using a rabbit antibody against *Campylobacter* (Dako, Glostrup, Denmark), or the bacteria were transformed with GFP-expressing plasmid pWM1007 [40]. For co-infection, we used the *E. coli* strain HB-101 expressing either the yellow fluorescent protein (YFP) from plasmid pWM1008 or the cyan fluorescent protein (CFP) from plasmid pWM1009 [40]. AlexaFluor-633-conjugated α-rabbit (#A-21070, Thermo Fisher Scientific, Waltham, MA, USA) secondary antibodies were employed to detect either α-occludin or α-*C. jejuni*. The nuclei and actin cytoskeleton were visualized using DAPI (4′-6-diamidino-2-phenylindole dihydrochloride) and rhodamine-phalloidin (R415, both from Thermo Fisher Scientific, Waltham, MA, USA), respectively. All samples were examined by confocal laser fluorescence microscopy using a Leica Stellaris 8 (Leica Microsystems, Wetzlar, Germany), and LAS AF computer software (Leica Microsystems Wetzlar, Germany) was used to visualize the obtained data at the Optical Imaging Centre Erlangen (OICE, Erlangen, Germany). To analyze the integrity of occludin patterns, the corresponding areas of tight junctions were segmented into the regions of interest (ROI) followed by the quantification of their fluorescence intensity using the Fiji platform, as described previously [21]. The relative fluorescence intensities were expressed as mean ± standard deviation (SD).

### 2.6. SDS-PAGE and Immunoblot Analysis

Protein samples derived from infected or uninfected cells were boiled in 1x SDS-PAGE buffer and loaded onto 10% SDS-PAGE gels, followed by blotting on PVDF Western blot membranes, as outlined previously [41]. Then, the membranes were blocked for 1 h using 5% skim milk in TBST buffer (0.1% Tween-20, 140 mM NaCl, 25 mM Tris–HCl pH 7.4, Carl Roth, Karlsruhe, Germany) at room temperature [42]. Mouse α-GAPDH antibodies were applied at 4 °C overnight according to the data sheet (Santa Cruz Biotechnology, Dallas, TX, USA). Horseradish peroxidase-conjugated α-mouse immunoglobulins were used as secondary antibodies (Thermo Fisher Scientific, Waltham, MA, USA). Finally, the ECL Prime chemiluminescence Western blot detection reagent (GE Healthcare, Chicago, IL, USA) was used to identify bound antibodies, as stated before [43].

### 2.7. Statistics

Each experiment was performed in triplicate. Evaluation of the data from immunofluorescence staining was performed by two-tailed Mann–Whitney tests, while the transmigration assays were examined by using one-way ANOVA followed by Tukey’s test with GraphPad Prism software (Version 8.0, GraphPad Software Inc., San Diego, CA, USA,). Statistical significance was defined by the obtained *p*-value (*p ≤* 0.0001 (****); n.s.—non significant).

## 3. Results and Discussion

### 3.1. C. jejuni Colonization of Apical Caco-2 Cell Surfaces

**Figure 1:**
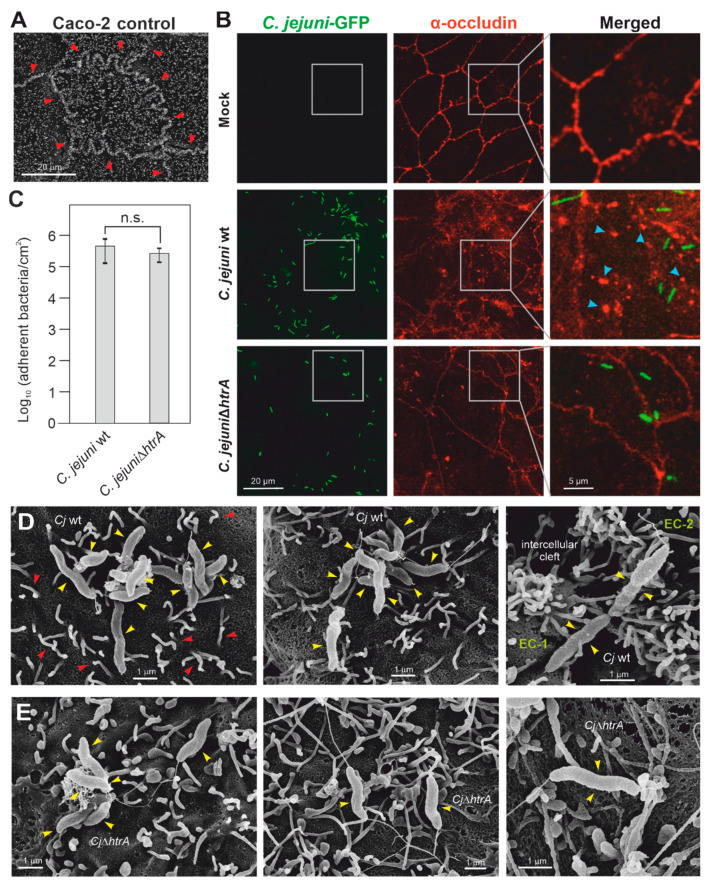
Scanning electron and fluorescence microscopy of Caco-2 cells before and after *C. jejuni* infection. (**A**) Top view of the apical surface of polarized Caco-2 cell monolayers without infection. Red arrowheads mark microvilli structures that are strongly expressed near the cell-to-cell junctions. (**B**) Fluorescence microscopy of Caco-2 cells counterstained with α-occludin (red), either as uninfected control (**top**), or infected with GFP-expressing wt bacteria (**middle**) or GFP-expressing ∆*htrA* mutant (**bottom**). The cells were infected with an MOI of 10. Images with the merged *C. jejuni* and occludin staining (**right**) correspond to enlarged areas marked in the gray boxes. Blue arrowheads indicate examples of disrupted patches in the tight junctions. (**C**) Quantification of apical bound bacteria revealed no significant (n.s.) differences among the two strains. Scanning electron microscopy of Caco-2 cells after infection with *C. jejuni* wt (**D**) or ∆*htrA* mutant (**E**). Attached *C. jejuni* bacteria are marked with yellow arrowheads and examples of microvilli with red arrowheads. Sporadically, infection with *C. jejuni* wt was seen with the local opening of cell-to-cell junctions visible as intercellular clefts formed at the apical cell surface between two neighboring epithelial cells (EC-1 and EC-2), as indicated.

To generate accurately polarized Caco-2 monolayers for our studies, it was necessary to let the cells differentiate for at least 14 days. During culturing, the functionality of the tight junctions was monitored every two days by phase contrast microscopy and by measuring the transepithelial electrical resistance (TER) in a transwell system. The overall TER values increased during this period until reaching maximal values between 350 and 400 Ohm × cm^2^, as described earlier [22]. In parallel, the cells were grown under identical conditions on glass slides placed in 12-well plates followed by analysis through scanning electron microscopy (SEM). The SEM technique identified the presence of characteristic cell-to-cell junctions and microvilli as apical markers (Figure 1A). The microvilli are the white-colored structures at the cell surface, which were enriched near the tight junctions (Figure 1A, red arrowheads, also exemplarily marked in image one of panel D). We then infected the Caco-2 cells with GFP-expressing *C. jejuni* wt and an available ∆*htrA* knockout mutant for 6 h, and investigated the bacteria bound apically to the Caco-2 cell surface by fluorescence microscopy (Figure 1B, green). The results show that the bacteria bound readily to the apical surface. Quantification revealed that *C. jejuni* wt and the ∆*htrA* mutant attached to the cells with similar numbers (Figure 1C). The cells were counter-stained using α-occludin antibodies to visualize proper tight junctions in the non-infected control cells (Figure 1B, red). In accordance with our former investigations [19,22], we found that *C. jejuni* wt infection induced the local disruption of cell-to-cell junctions visible as occludin aggregates formed at the apical cell surface (Figure 1B, blue arrowheads), while this phenotype was widely diminished during infection with the ∆*htrA* mutant (Figure 1B, bottom). Tight bacterial attachment to the apical surface was then confirmed by SEM, as observed for both wt (Figure 1D) and ∆*htrA* mutant *C. jejuni* (Figure 1E). However, signs of apical bacterial invasion into the host cell cytoplasm were not evident. In some cases, surface-bound wt bacteria were found near the cell–cell junctions, which formed local “clefts” between neighboring epithelial cells (Figure 1D, right panel). In contrast, the ∆*htrA* mutant bound non-specifically to the apical cell surface without obviously affecting the cell-to-cell junctions (Figure 1E, yellow arrowheads).

### 3.2. Colonization of Caco-2 Cells by C. jejuni and E. coli, and Impact on Tight Junctions

**Figure 2:**
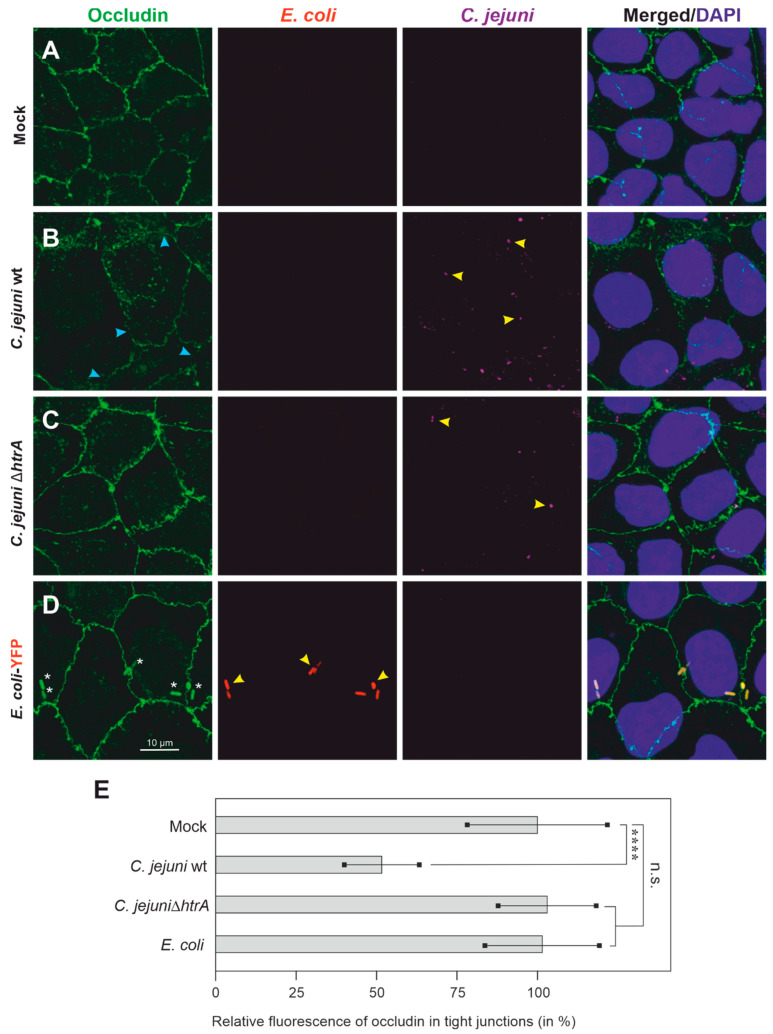
Top view of infected Caco-2 cells by immunofluorescence microscopy. Epithelial cell monolayers were left uninfected (Mock, **A**) or were infected with either *C. jejuni* wt (**B**), *C. jejuni* Δ*htrA* (**C**) or *E. coli*-YFP (**D**). After infection with an MOI of 10, the cells were immunostained using α-occludin and α-*C. jejuni*-specific antibodies as well as DAPI. Yellow arrowheads indicate bacterial cells interacting with Caco-2 cells at their apical surface and blue arrowheads show disrupted tight junctions. The white stars (panel **D**, left) indicate non-specific fluorescence signals from *E. coli*-YFP. (**E**) Relative fluorescence intensities of occludin in the tight junction areas were quantified and presented as the mean ± SD. The mean relative fluorescence of uninfected mock cells was set as 100%. Significant differences shown in the graphs correspond to *p* ≤ 0.0001 (****) or non-significant (n.s.).

We have previously demonstrated that *C. jejuni* can colonize polarized epithelial cell monolayers and affect junctional proteins by HtrA-dependent cleavage, thus disrupting the epithelial barrier [19,21,22]. Next, we wanted to investigate whether such properties may impact further microorganisms, such as non-invasive *E. coli*. In a first experiment, we explored if polarized Caco-2 cells are suitable to address this question. Therefore, Caco-2 epithelial cells were cultured as described above, then infected with the two *C. jejuni* strains or YFP-expressing *E. coli* and subjected to immunofluorescence (Figure 2). Examples of bacteria bound to the apical Caco-2 cell surface are marked with yellow arrowheads. The immunofluorescence pictures show that *C. jejuni* wt adhered to the cell surface and induced the disruption of the cellular tight junction component occludin, as expected (Figure 2B, green, blue arrowheads). Infection with *C. jejuni* Δ*htrA* mutant or *E. coli*-YFP showed that these bacteria also adhered to the cell surface, but the occludin-containing cellular tight junctions were not disrupted (Figure 2C,D, green). The corresponding quantification data are displayed in Figure 2E.

### 3.3. Confocal Microscopy of Co-Transmigrating C. jejuni and E. coli across Polarized Cells

**Figure 3:**
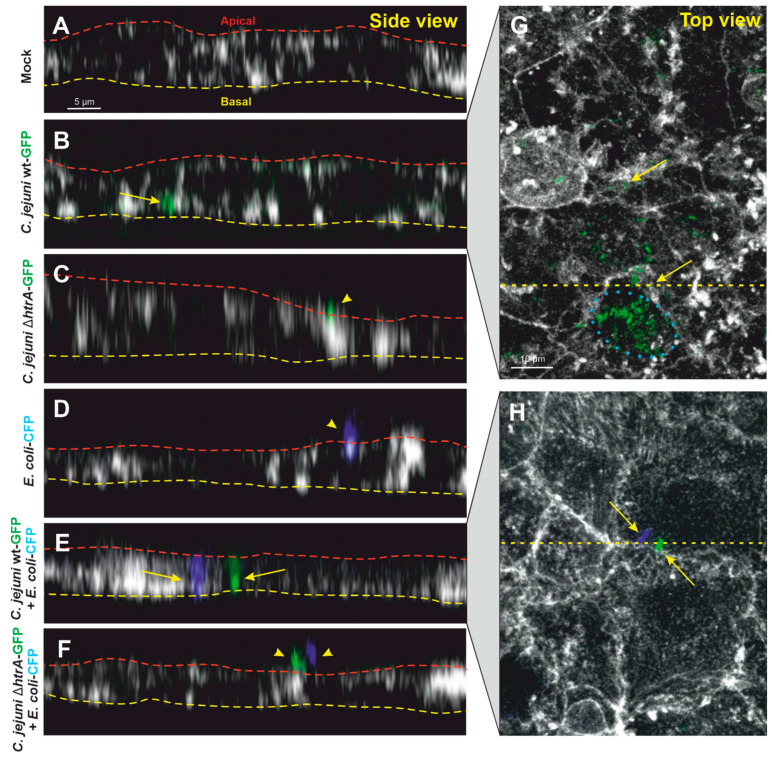
Side and top view of infected Caco-2 monolayers by confocal fluorescence microscopy. The cells were infected with the indicated *C. jejuni* and/or *E. coli* bacteria and counterstained with rhodamine-phalloidin to visualize the actin cytoskeleton (gray). Lateral sections (Z-orientation) are given for showing bacterial transmigration to basolateral sides of epithelial cells. Caco-2 monolayers were left uninfected (**A**) or infected with either *C. jejuni*-GFP wt (**B**), *C. jejuni* Δ*htrA*-GFP (**C**) or *E. coli*-CFP (**D**). Dual co-infections were performed using wt *C. jejuni*-GFP and *E. coli*-CFP (**E**) or *C. jejuni* Δ*htrA*-GFP and *E. coli*-CFP (**F**). The red and yellow dashed lines mark apical and basal sides of the cell monolayers, respectively. The yellow arrowheads indicate attached bacterial cells, and yellow arrows mark transmigrating bacteria from apical to basal sides. (**G**,**H**) Top view of enlarged sections showing the localization of transmigrating bacteria that are between neighboring cells at the basal side (yellow arrows). The images also show some intracellular *C. jejuni.* Examples are marked with a blue dotted circle. The yellow dashed lines indicate the corresponding cross sections. The cells were infected with an MOI of 1 to detect single bacterial cells.

We have previously shown that transmigration of *C. jejuni* across polarized epithelial cell monolayers is enabled by cleaving occludin and other junctional proteins in an HtrA-dependent manner [19,21,22]. Next, we wanted to examine if commensal bacteria such as *E. coli* can travel together with *C. jejuni* across polarized Caco-2 epithelial cells. For this purpose, we monitored the transmigration of *C. jejuni* and *E. coli* (either alone or together) by confocal microscopy (Figure 3). Indeed, wt *C. jejuni*-GFP alone could be detected between two neighboring cells or at the basal side of the infected Caco-2 monolayer (Figure 3B), while *C. jejuni* Δ*htrA*-GFP or *E. coli*-CFP alone were only found on the apical cell surface (Figure 3C,D). However, when Caco-2 cells were co-infected with wt *E. coli*-CFP and *C. jejuni*-GFP, both bacterial species could be observed basolaterally between neighboring epithelial cells in close proximity to each other (Figure 3E). In contrast, when Caco-2 cells were co-infected with *E. coli*-CFP and *C. jejuni* Δ*htrA*-GFP, both bacterial species were unable to transmigrate and could be found only on the apical monolayer surface (Figure 3F). In both infections, *C. jejuni* wt alone or co-infection with *E. coli*, bacterial cells were detected in close proximity to the tight junctions (enlarged sections in Figure 3G,H). Together, these findings underline the importance of HtrA in paracellular transmigration of *C. jejuni*, which at the same time can trigger the transmigration of non-invasive *E. coli*. In addition, we sporadically detected some intracellular *C. jejuni* wt (Figure 3G, blue dotted circle) that probably invaded the cells from the bottom via interaction of CadF with the basal fibronectin-integrin complex [13].

### 3.4. Quantification of Co-Transmigrating C. jejuni and E. coli across Polarized Caco-2 Cells

**Figure 4:**
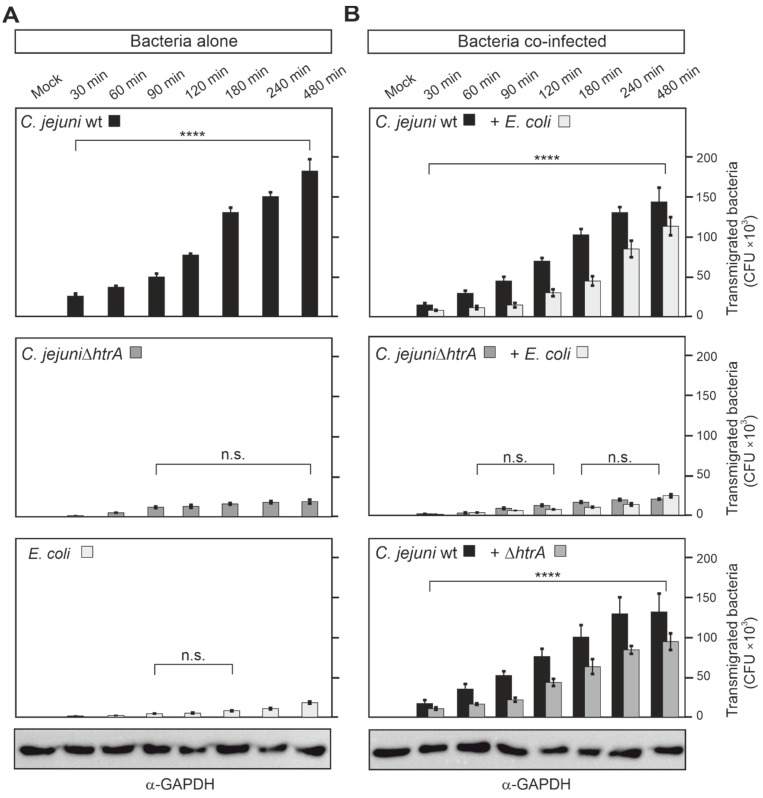
Time course of transmigration properties of *C. jejuni* and *E. coli* bacteria across polarized Caco-2 cells using a transwell filter system. Caco-2 cells were cultivated for 14 days to form polarized monolayers on transwell filters and then infected with the indicated single bacterial strains (**A**) or mixed bacterial strains (**B**) at an MOI of 25 each. Transmigrated bacteria were collected from the bottom chambers, and *C. jejuni* were cultivated on *Campylobacter* agar plates and *E. coli* on LB agar plates, respectively. Finally, the CFUs were quantified in triplicate. On the bottom are two representative GAPDH blots as loading controls. Significant differences shown in the graphs correspond to *p* ≤ 0.0001 (****) or non-significant (n.s.).

Next, our aim was to quantify the transmigration rates of *C. jejuni and E. coli.* For this reason, Caco-2 cells were cultivated on transwell inserts and differentiated as described above. Then, infection of the cells was performed by adding the bacteria to the apical chamber, and the numbers of transmigrating bacterial CFUs in the bottom chamber were quantified in a time course up to 8 h (Figure 4). In agreement with the findings we acquired from confocal microscopy, we found that *C. jejuni* wt transmigrated fast and effectively. The first transmigrated bacteria were even seen after 30 min, and up to 200,000 transmigrated bacteria at the 8 h time-point (Figure 4A). In contrast, both the Δ*htrA* deletion mutant and *E. coli* alone revealed a robust deficiency in transmigration in comparison to *C. jejuni* wt (Figure 4A). When Caco-2 cells were co-infected with *C. jejuni* wt and *E. coli*, but not *C. jejuni* Δ*htrA* with *E. coli,* both bacterial species transmigrated effectively (Figure 4B, top). After 8 h of infection, the number of transmigrated *E. coli* significantly increased (adjusted *p*-value less than 0.0001) from about 18,000 in Caco-2 cells infected alone to 113,000 in cells co-infected with *C. jejuni* wt and *E. coli*. Interestingly, when Caco-2 cells were co-infected with *C. jejuni* wt and *C. jejuni* Δ*htrA*, the transmigration deficiency of the mutant was rescued (Figure 4B, bottom). The number of transmigrated Δ*htrA* mutants significantly increased (adjusted *p*-value less than 0.0001) from about 19,000 when added to Caco-2 cells alone to 94,000 when added to the cells concurrently with *C. jejuni* wt. Together, these findings underline the importance of HtrA in paracellular transmigration of *C. jejuni*, which at the same time can trigger the transmigration of non-invasive *E. coli* or HtrA-defective *C. jejuni*.

### 3.5. Epithelial Transmigration of Other Microbiota by HtrA-Expressing C. jejuni

**Figure 5:**
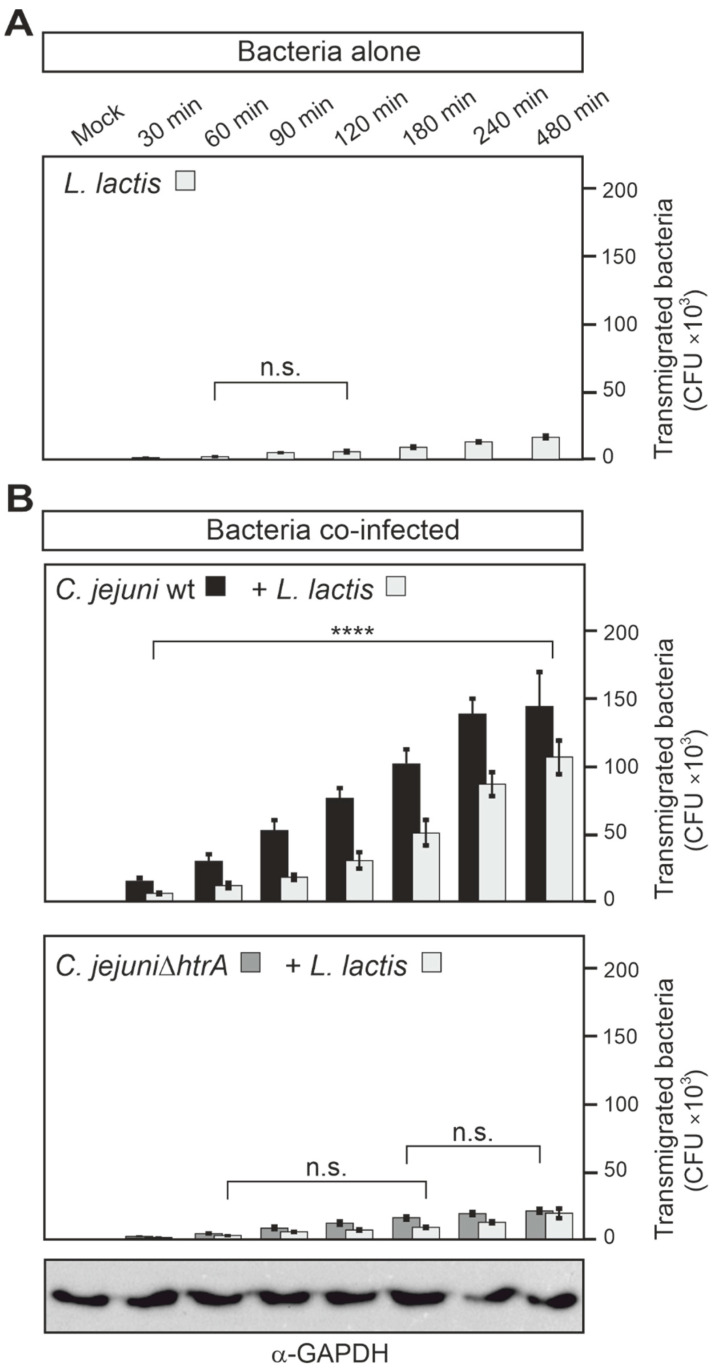
Time course of transmigration properties of *C. jejuni* and *L. lactis* bacteria across polarized Caco-2 cells using a transwell filter system. Caco-2 cells were cultivated for 14 days to form polarized monolayers on transwell filters and then infected with the indicated single *L. lactis* strain (**A**) or mixed bacterial strains (**B**) at an MOI of 25 each. Transmigrated bacteria were collected from the bottom chambers. *C. jejuni* were grown on *Campylobacter* agar plates and *L. lactis* on MRS agar plates. Finally, the CFUs were quantified in triplicate. On the bottom is a representative GAPDH blot as a loading control. Significant differences shown in the graphs correspond to *p* ≤ 0.0001 (****) or non-significant (n.s.).

Finally, we wanted to explore if other microbiota bacteria can transmigrate across the intestinal epithelium in the presence of *C. jejuni*. We therefore tested *Lactococcus lactis.* This commensal bacterium alone showed weak transmigration capabilities, similar to non-pathogenic *E. coli* (Figure 5A). However, when Caco-2 cells were co-infected with *L. lactis* and *C. jejuni* wt, but not *C. jejuni* Δ*htrA* and *L. lactis,* both bacterial species transmigrated effectively (Figure 5B). After 8 h of infection, the transmigration rate of *L. lactis* significantly increased (adjusted *p*-value less than 0.0001) from about 16,000 bacteria in Caco-2 cells when infected alone to 106,000 *L. lactis* bacteria when co-infected with *C. jejuni* wt. These observations confirm that secreted HtrA of *C. jejuni* has a crucial function in crossing the epithelial barrier by this pathogen, as well as commensal bacteria of the microbiota such as *E. coli* or *L. lactis*.

## 4. Conclusions

The epithelium in the digestive tract of humans represents an effective barrier, protecting the host against intruding microorganisms such as microbiota. Various gastrointestinal microbial pathogens, including Helicobacter, Shigella, Salmonella, Listeria and others, exhibit cell-invasive capabilities and damage the epithelial barrier, followed by transcellular or paracellular migration [15,44,45]. Earlier reports have shown that *C. jejuni* is able to transmigrate across polarized MKN-28, Caco-2 and T84 epithelial cell monolayers in vitro via the transcellular route [8,33] or paracellular route [34], but a final consensus is still not found in the community. However, Campylobacter infection represents the most common risk factor for developing IBD during the first year after campylobacteriosis. Patients with IBD possess pronounced inflammatory reactions to their intestinal microbiota by mechanisms that are not fully understood [29]. In the healthy intestine, the microbiota is successfully segregated to the lumen by the intestinal epithelium. Nevertheless, defects of the intestinal epithelial barrier by dysregulated cytokines/chemokines and disrupted tight junctions, known as the “leaky gut” phenotype, may contribute to IBD development, because bacteria can now translocate across the epithelium to the lamina propria. In this way, they can expose their antigens to submucosal immune cells and provoke an inflammatory reaction against the normal microbiota [28]. This scenario can trigger or aggravate inflammatory reactions in IBD patients.

*C. jejuni* infection has been known to disturb the intestinal barrier integrity [2,45,46]. Our recent data showed that *C. jejuni* serine protease HtrA can be secreted in the extracellular space, where HtrA cleaves the junctional proteins E-cadherin, occludin and claudin-8 [18,19,20,21,22,35]. Here, we demonstrate that *C. jejuni* bind to the apical surface of polarized Caco-2 cells, but do not invade into the cytoplasm from the apical side. Instead, they loosen the cell-to-cell junctions, and rapidly cross the epithelial monolayer between neighboring cells in an HtrA-dependent fashion. Moreover, we could demonstrate that in this scenario, microbiota strains such as non-invasive *E. coli* or *L. lactis* also translocate by the same route into the basal compartment in a time-dependent fashion. Remarkably, *C. jejuni* wt, but not ∆*htrA* mutant, induced the efficient transmigration of these microbiota bacteria. We therefore suggest that the microbiota strains, which all encode *htrA* genes, may express HtrA proteins that are different from *C. jejuni* and cannot cleave host junctional proteins. Taken together, *C. jejuni* infection of the intestinal epithelium results in local opening of cellular junctions and paracellular translocation in an HtrA-dependent manner, associated with co-transmigration of microbiota. This mechanism may explain how microbiota strains can traverse the gut epithelium with the help of *C. jejuni.* In this way, microbiota can establish contact with host immune cells and provoke undesired immune responses. The exact translocation procedure, however, is not yet clear and should be studied in future experiments. Nevertheless, these findings may have an important impact on our understanding of the molecular mechanism behind various *Campylobacter*-associated diseases such as IBD. Thus, *C. jejuni* HtrA could be a promising new anti-microbial target for therapy in IBD patients.

## Data Availability

The data that support the findings of this study are available from the corresponding author upon request.
